# Three-dimensional tonotopic mapping of the human cochlea based on synchrotron radiation phase-contrast imaging

**DOI:** 10.1038/s41598-021-83225-w

**Published:** 2021-02-24

**Authors:** Hao Li, Luke Helpard, Jonas Ekeroot, Seyed Alireza Rohani, Ning Zhu, Helge Rask-Andersen, Hanif M. Ladak, Sumit Agrawal

**Affiliations:** 1 Department of Surgical Sciences, Section of Otolaryngology, Department of Otolaryngology, Uppsala University Hospital, 751 85 Uppsala, Sweden; 2 School of Biomedical Engineering, Western University, 1152 Richmond St, London, ON N6A 3K7 Canada; 3 Department of Otolaryngology, Head and Neck Surgery, Western University, London, ON Canada; 4 Bio-Medical Imaging and Therapy Facility, Canadian Light Source Inc., University of Saskatchewan, Saskatoon, SK Canada; 5 Department of Medical Biophysics, Western University, London, ON Canada; 6 Department of Electrical and Computer Engineering, Western University, London, ON Canada

**Keywords:** Anatomy, Health care, Medical research, Neurology, Physics

## Abstract

The human cochlea transforms sound waves into electrical signals in the acoustic nerve fibers with high acuity. This transformation occurs via vibrating anisotropic membranes (basilar and tectorial membranes) and frequency-specific hair cell receptors. Frequency-positions can be mapped within the cochlea to create a tonotopic chart which fits an almost-exponential function with lowest frequencies positioned apically and highest frequencies positioned at the cochlear base (Bekesy 1960, Greenwood 1961). To date, models of frequency positions have been based on a two-dimensional analysis with inaccurate representations of the cochlear hook region. In the present study, the first three-dimensional frequency analysis of the cochlea using dendritic mapping to obtain accurate tonotopic maps of the human basilar membrane/organ of Corti and the spiral ganglion was performed. A novel imaging technique, synchrotron radiation phase-contrast imaging, was used and a spiral ganglion frequency function was estimated by nonlinear least squares fitting a Greenwood-like function (*F* = *A *(10^*ax*^ − *K*)) to the data*.* The three-dimensional tonotopic data presented herein has large implications for validating electrode position and creating customized frequency maps for cochlear implant recipients.

## Introduction

The human cochlea is a spiral structure of the inner ear contained within the temporal bone, the densest bone in the body. To produce the sensation of hearing, sound vibrations are transmitted to the cochlea and transduced into electrical activity along the basilar membrane (BM). The BM is a soft-tissue structure which spatially separates acoustic vibrations based on frequency, resulting in a spatial cochlear frequency map. This spatial distribution of frequencies is referred to as cochlear tonotopy. Nerve fibers traverse from the BM to the spiral ganglion (SG), a structure closer to the axis of rotation of the cochlear spiral which contains neuron cell bodies.

The delicate tissue and sensory cells responsible for this mechanoelectrical transduction in the inner ear are at a high risk of damage, which can lead to sensorineural hearing loss. This sensory cell damage (and associated sensorineural hearing loss) can result from genetic causes, birth complications, infections, certain drugs, noise exposure, and with age. In the Global Burden of Disease Study, hearing loss was found to be the fourth leading cause of disability; higher than diabetes, dementia, and chronic obstructive pulmonary disease^1^. According to the World Health Organization^2^, 466 million people worldwide have disabling hearing loss, 34 million of which are children. In recent years, there has been remarkable progress in the treatment of severe and profound sensorineural hearing loss with cochlear implantation (CI) both in children and adults. In CI, electrode arrays are inserted into the cochlea to directly stimulate the nerve fibers with electrical signals, thereby by-passing the failing sensory cells and mechanoelectrical transduction. CI has become the preferred method for treating many individuals with hearing loss, including children born with deafness. Additionally, individuals with hearing loss but with some remaining low-frequency hearing can be treated with CI to conserve their residual hearing.

Accurate tonotopic stimulation, meaning cochlear implant electrodes stimulate according to their post-operative tonotopic locations, is expected to provide the best functional outcome for CI patients^3–5^. Detailed measurements of anatomical structures such as the BM and SG can aid in the understanding of how cochlear frequency maps vary between individuals, however accurate visualization of these structures is not possible using common clinical imaging modalities such as computed tomography (CT) and magnetic resonance imaging (MRI). Previous measurements of cochlear anatomy have heavily relied on histological analysis, however these techniques have inherent artefacts and spatial limitations, often related to the decalcification, sectioning, and staining of the specimens^6,7^. Stakhovskaya et al*.*^8^ published histologically-derived SG tonotopy measurements, which have become the most widely accepted and utilized information for SG frequency mapping. This previous work was based on two-dimensional histological sections and has not been validated in three-dimensions.

In this study, the first detailed three-dimensional (3D) measurements of the BM and SG are presented using synchrotron radiation phase-contrast imaging (SR-PCI). This imaging technique has the advantage of simultaneous bone and soft tissue visualization without the need for sectioning and staining^9^. Using SR-PCI, it is possible to visualize cellular level information^9^ in the cochlea, in addition to making 3D geometric measurements of complex anatomy such as the helicotrema^10^ and hook region. The objective of the present work is to use manual dendrite tracing in SR-PCI data to determine accurate 3D tonotopic coordinates of the human SG.

## Material and methods

### Human temporal bones and ethics

The study was approved by Western University, London, Ontario, Canada in accordance with the Anatomy Act of Ontario and Western’s Committee for Cadaveric Use in Research (approval no. 06092020). Ten fixed adult human cadaveric cochleae were used in this study. Specimens were obtained with permission from the body bequeathal program at Western University, London, Ontario, Canada in accordance with the Anatomy Act of Ontario and Western’s Committee for Cadaveric Use in Research (approval no. 06092020). All methods were carried out in accordance with relevant guidelines. No staining, sectioning, or decalcification were performed on the specimens.

### Imaging technique

The SR-PCI technique used in the present investigation was recently described by Elfarnawany et al.^11^ and Koch et al.^12^. Each sample was scanned using SR-PCI combined with CT at the Bio-Medical Imaging and Therapy (BMIT) 05ID-2 beamline at the Canadian Light Source, Inc. (CLSI) in Saskatoon, SK, Canada. The imaging field of view was set to 4000 × 950 pixels corresponding to 36.0 × 8.6 mm, and 3000 projections over a 180° rotation were acquired per CT scan. Computed tomography reconstruction was performed, and the 3D image volume had an isotropic voxel size of 9 μm. The acquisition time to capture all projections per view was ~ 30 min.

### 3D Reconstruction and construction of frequency maps

For 3D reconstructions of the BM, SG, and other cochlear anatomy, structures were traced and color-labeled manually on each SR-PCI CT slice (approximately 1400 slices per sample). The open source medical imaging software, 3D Slicer (www.slicer.org, version 4.10)^13^, was used to create detailed 3D representations of the BM, SG, and connective dendrites between these structures, which allowed for accurate delineation when compared to traditional two-dimensional (2D) slices. Dendrites were individually traced from the BM to the SG at each frequency coordinate in three-dimensions, allowing for 3D analysis of SG tonotopy. The BM tonotopic frequency at each dendrite location was calculated using Greenwood’s function^14^, which relates proportional length along the BM to characteristic frequency. Individually tracing the BM, SG, and connective dendrites in three-dimensions allowed for precise tonotopic representations of both the BM and SG, especially in areas such as the hook region and helicotrema where the dendrites follow a non-radial trajectory from the BM to the SG. Since the spatial resolution of SR-PCI is coarser than light microscopy using the present fixation and non-staining, cellular details within the SG were occasionally challenging to discern. However, dendrites tracing from the BM were visible, along with Rosenthal’s canal (the bony conduit containing the SG), which has previously been analyzed in SR-PCI data^15^.

Angular frequency coordinates were additionally obtained for the BM and SG in all samples by measuring the angular depth of each manually traced dendrite connection and corresponding tonotopic frequency. A reformatted image slice was obtained through the mid-modiolar axis of each cochlea in three-dimensions, and this reformatted image slice was rotated about the mid-modiolar axis through the length of the BM and SG. With 0 degrees defined as the center of the round window (RW), the angular depth of each frequency coordinate along the BM and SG was obtained. Manually measured angle-frequency coordinates were interpolated to obtain a set of coordinates at common intervals across all samples for the purposes of inter-sample averaging and comparison.

To mathematically describe the 3D SG tonotopic coordinates obtained herein, a non-linear least-squares fit was performed to determine a Greenwood-like exponential function which relates percentage length along the SG to tonotopic frequency. Although percentage distances along the SG are generally not obtainable measurements in clinical or research imaging modalities, an exponential function was fit to the SG tonotopic coordinates to provide an anatomical description and to allow for mathematical comparison between BM and SG distributions.

## Results

**Figure 1:**
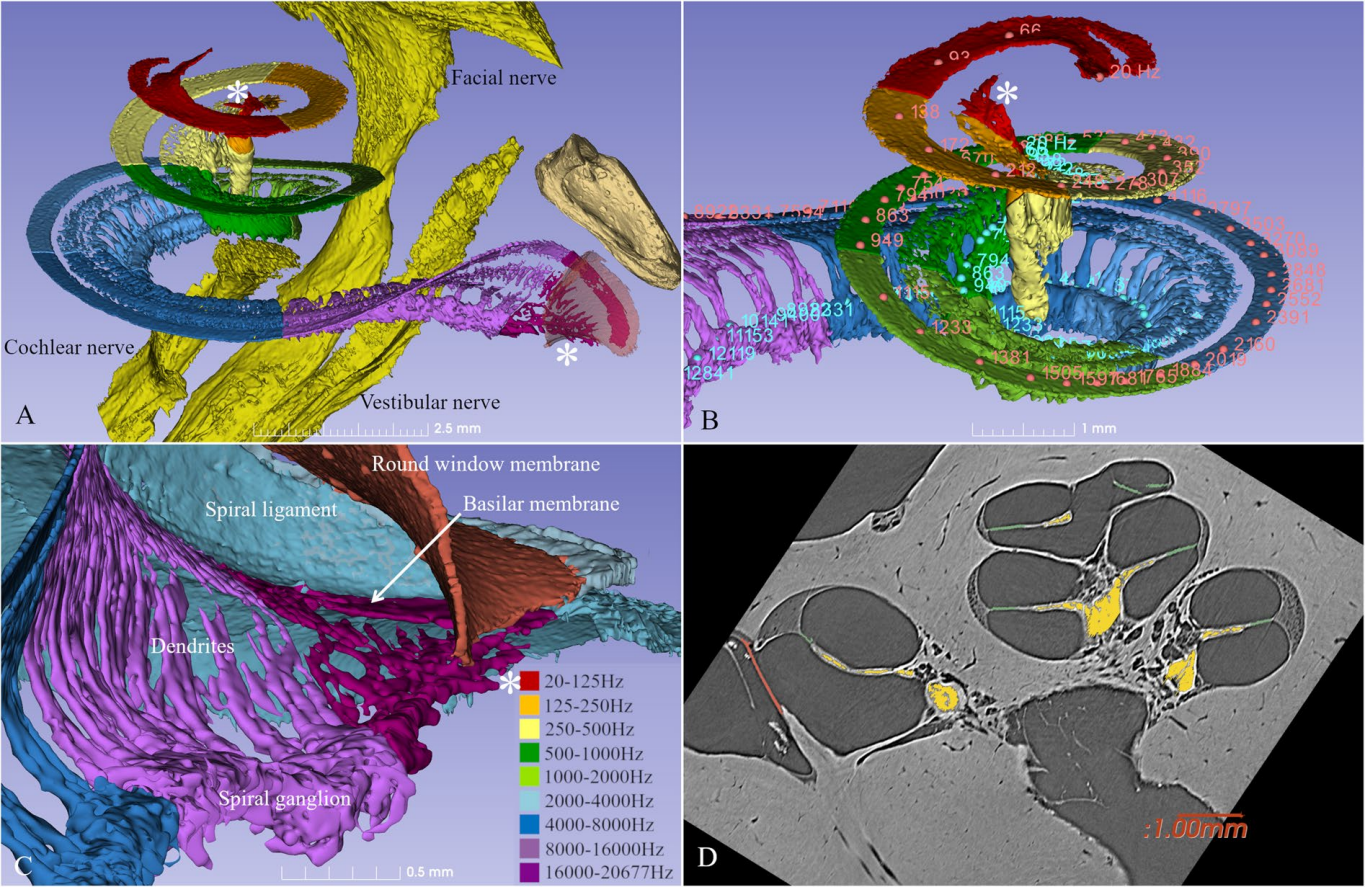
(**A**) SR-PCI data of a left human cochlea. 3D Slicer (www.slicer.org, version 4.10.1)^13^ was used to create a detailed 3D representation including intra-cochlear soft tissue. The basilar membrane and spiral ganglion were segmented, and the frequency coordinates were calculated using Greenwood’s formula^14^ and dendrite tracing. (**B**,**C**) For the spiral ganglion, the dendrites were traced from the basilar membrane to make a corresponding frequency map (shown with color scale). Note the angle of dendritic connections are not radial to the mid-modiolar axis in the apical and basal region (denoted by *). (**D**) Representative tomographic X-ray section showing the segmented round window (red), neural elements (yellow) and basilar membrane (green). GIMP 2 (www.gimp.org) was used to create the figures.

**Figure 2:**
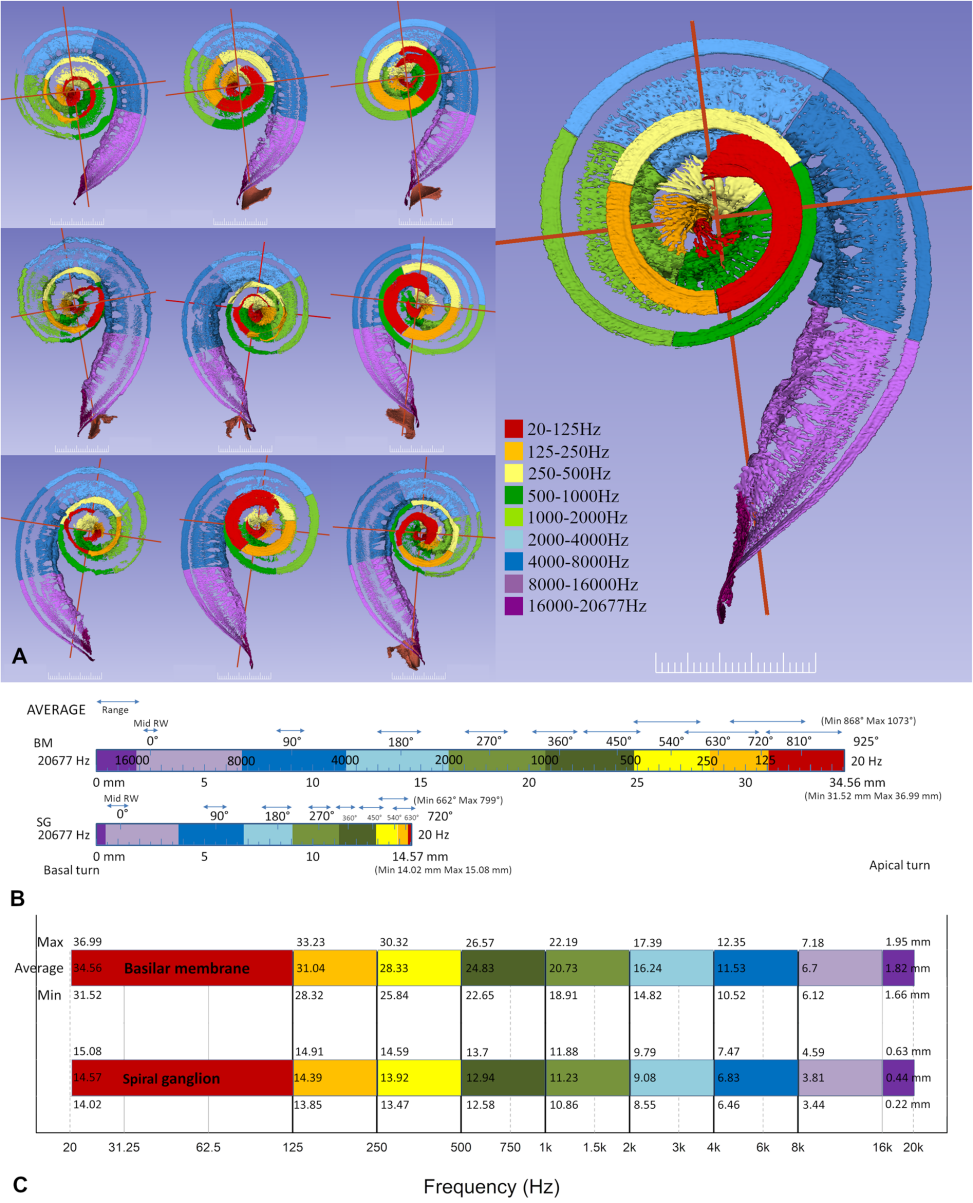
(**A**) Segmentations of soft tissues from ten cochleae shown in orthographic 3D view. 3D Slicer (www.slicer.org, version 4.10.1)^13^ was used to create the 3D representations. Frequency maps of the basilar membrane were developed according to Greenwood^14^. Corresponding dendrites were traced to the spiral ganglion and corresponding octave bands are outlined. Scale bar is 2.5 mm. (**B**) Average rotational angles for tonotopic frequencies of the BM and SG. Angular rotations are calculated from the mid-point of the round window. Ranges in position of angular rotations are shown with blue arrows. (**C**) Average length of BM and SG presented against frequency for all cochleae. Values within the color bars represent the mean distance to reach each tonotopic frequency, with values along the top and bottom of the color bars representing the maximum and minimum distances to reach each frequency, respectively.

**Figure 3:**
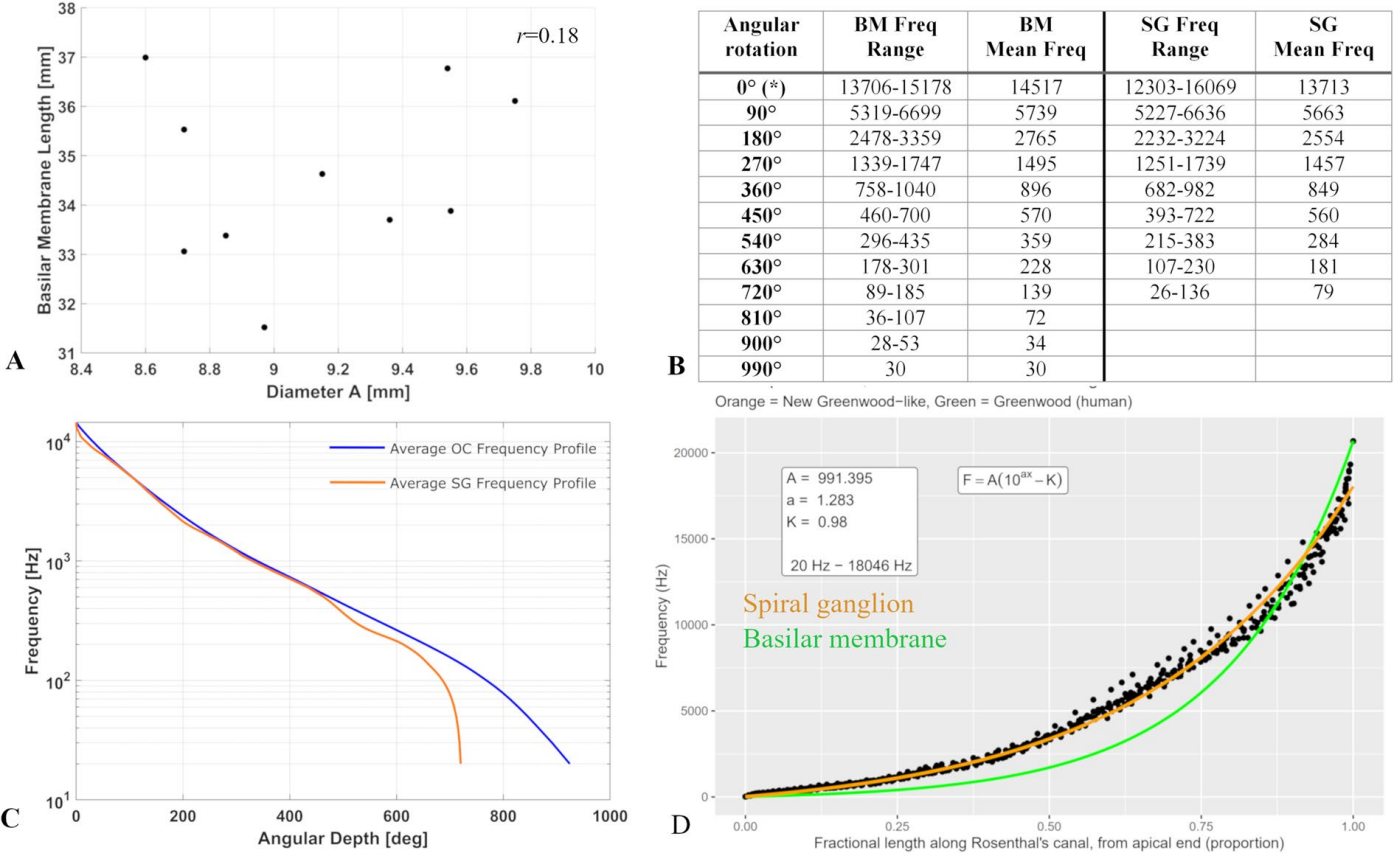
(**A**) Basilar membrane length is plotted against diameter *A*. No correlation was noted between these values (*r* = 0.18). (**B**) Table showing mean angular frequencies obtained from ten specimens imaged using synchrotron radiation phase-contrast imaging. (**C**) Average basilar membrane and spiral ganglion frequencies plotted against cochlear angular depth. This average curve was determined by calculating the mean angular depth of each frequency in all samples. (**D**) Results of least squares fitting an exponential function to 3D SR-PCI SG tonotopic data. Greenwood’s function for the BM tonotopic distribution is illustrated in green for reference, and the SG function relating proportional length to tonotopic frequency developed herein is illustrated in orange.

Three-dimensionally rendered structures derived from SR-PCI images of one human cochlea are shown in Fig. 1A–C. The intra-cochlear soft tissues were modeled, and the tonotopic coordinates of the BM and SG based on dendritic mapping are presented with octave bands. One SR-PCI image slice of the cochlea is shown in Fig. 1D. The mean length of the BM was 34.6 mm (range 31.5–36.9 mm) and the mean length of the SG was 14.6 mm (range 14–15.1 mm). Angular frequency coordinates of the BM and SG are presented in Figs. 2 and 3. The mean angular length from the center of the RW was 925° (range 868°–1073°) for the BM and 720° (662°–799°) for the SG. The mean frequency locations in all ten modeled cochleae are shown in Fig. 2A. Both the shape and dimensions of the cochleae were observed to vary, and this influenced the spatial location of tonotopic frequencies and octave bands. The total length and extensions of various octave bands of the BM varied considerably compared to those of the SG.

The diameter *A* is defined as the long diameter of the basal, first turn of the cochlear spiral, measured from round window to lateral wall. This diameter is frequently used to estimate the cochlear length, indicating that the diameter *A* may occasionally underestimate cochlear length and therefore result in inaccurate estimations of BM tonotopic coordinates. In this series, the cochlea having the longest BM (37 mm), actually had the shortest diameter A (8.6 mm, Fig. 3A, Supplementary Fig. 1). The diameter *A* is plotted in comparison to BM length in Fig. 3A. A very weak correlation was observed between the diameter *A* and the complete BM length (r = 0.18), which is consistent with the prior analysis by Erixon and Rask-Andersen^16^, which demonstrated an R2 value of 0.39. Although the prior analysis revealed a higher correlation for the basal turn and two-turn length, there are potential consequences associated with predicting the total BM length using solely the diameter *A* due to the apical turn variability. Interestingly, we see here among the ten specimens some unusual anatomic variants where the cochlear coiling was different. These specimen are additional reasons for the low/absent correlation here. Excluding these two specimen gives a much higher correlation.

Average tonotopic frequencies of angular depths are shown in Fig. 2B. The results from all ten specimens are shown separately in Supplementary Fig. 1. Figure 2C demonstrates the variations in length of the different octave bands projected in a full-spectrum audiogram. The mean length of the 16,000–20,677 Hz frequency band was 1.8 mm for the BM and 0.4 mm for the SG. This represents 5% of the entire BM length and less than 3% of the SG length. The most basal part of the SG was narrow and contained cells innervating the BM surrounding the RW membrane^17^. The length of the BM octave bands decreased from base to apex and this decrement was more pronounced for the SG. The mean length of the 8–16 kHz octave band was 4.9 mm for BM and 3.4 mm for SG.

The shape and course of the dendrite columns and neural branching varied along the cochlear spiral. Dendrites had a direct radial course in the first turn except at the RW. Apically, the dendrites started to rotate to a non-radial trajectory near the 700 Hz region, corresponding to approximately 400 degrees of angular depth.

The tonotopic frequency ranges of the BM and SG at discrete angular depths are presented in tabular form in Fig. 3B. The average BM and SG angular frequency profiles are illustrated in Fig. 3C. The result of fitting an exponential function to the SG tonotopic distribution is shown in Fig. 3D.

3D model of SG, BM and dendrites are publicly accessible through Auditory Biophysics Laboratory website (https://abl.uwo.ca/).

## Discussion

In the present study, SR-PCI data was used to obtain detailed 3D reconstructions of the human BM, SG and cochlear dendrites. In 1999, Vogel^18^ published work using absorption-based X-ray CT to image the human inner ear, in which a spatial resolution in the range of 10 μm was achieved. Although synchrotron radiation was used by Vogel, the absorption-based contrast did not provide adequate soft tissue discernment in structures such as the BM. Lareida et al*.*^19^ and Muller et al*.*^20^ used 1% OsO_4_ in 0.05 M cacodylate buffer staining to visualize structures such as the organ of Corti and nerve fiber bundles using absorption based X-ray CT with a synchrotron source. The ability to discern structures with staining was a significant improvement, however the RW and OW were opened during sample preparation, and artefacts have been reported to arise from the staining of soft tissues^6,21^. Rau et al*.*^22^ utilized SR-PCI to visualize fine structures such as organ of Corti hair cells in gerbil cochleae, however 3D reconstructions were not presented. Elfarnawany et al*.*^11^ were the first to perform SR-PCI on intact human cochleae, and obtained 3D reconstructions of cochlear soft tissues and achieved visualization of cytoarchitecture^9^. In PCI, the phase shifts caused by varying material properties within the sample are transformed into detectable variations in x-ray intensity, which can provide edge contrast to highlight soft tissues. Phase contrast is therefore combined with synchrotron radiation to improve soft-tissue contrast while maintaining accurate visualization of bone^11^, while avoiding the artefacts introduced with staining, sectioning, and decalcification used in histopathology^6^.

Stakhovskaya et al*.*^8^ previously published a human SG tonotopic map which was large advancement in the field. The work, however, was limited by the histological approach used. In order to trace dendrites between the BM and SG, Stakhovskaya et al*.* prepared histological sections which resulted in measurements that are limited to 2D. The present study is the first to perform 3D reconstruction and dendrite tracing of intact human cochleae, allowing for accurate tonotopic mapping of the BM and SG through fully attached complex anatomy. Accurate 3D visualization is particularly important in regions such as the cochlear hook, where dendrites follow a non-radial trajectory and tonotopic compression is observed in the SG.

The imaging and anatomical reconstruction techniques applied in the present study were recently used by Li et al*.*^15^ to study SG. The SG was observed to extend approximately two turns relative to the BM, which is consistent with the description by Guild et al*.*^23^. The present results also agree with the computer-based 3D reconstruction based on serial celloidin sections performed by Ariyasu et al*.*^24^. Moreover, histological analyses by Stakhovskaya et al*.*^8^ found that the SG extends 720° when measured from the start of the RW and a frequency map with rotational data was established^8,25^. These amassed results contrast to the one and half turn description by Nadol and Merchant^26^, who separated four SG regions where almost one third of all neurons were located in the upper half of the SG. According to Guild et al.^23^, the most densely innervated portion of the hearing organ is the upper basal and neighboring lower middle turn. This is consistent with the present findings.

The present 3D modelling shows that SG length ranges from 14.02 to 15.08 mm with octave band dimensions and distances from the base having low variability relative to the BM. The SG ends with a thickening or apical bulb that is crowded with neurons^27^. Here, the frequency bands are narrow, where the four octave bands below 1 kHz are all located within the apical 3.4 mm of the SG (Fig. 2C). These compressed neurons may constitute an obstacle for selective place stimulation of low-frequency neurons by a cochlear implant. From an anatomical standpoint, optimal reach of the SG would seem to be at two turns, thereby covering the entire frequency range. In the present model, the distance from the RW to this point was 14–15 mm and showed little variability. The corresponding length to reach the BM at two turns was around 31 mm and showed greater variability. Due to individual variability in cochlear shape, the stimulation pattern of peripheral neural components^16,28–33^ and the BM may be significantly influenced. The ranges of frequency band dimensions increased basally for both the SG and BM (Supplementary Fig. 1). In the SG, tonotopic frequencies were observed to be spread over a shorter length (both linear and angular) compared to the BM. This compression of frequencies is particularly prominent in the low frequency cochlear apex (Fig. 3C) where dendrites from a large region of the BM map to a narrow portion on the SG. Therefore, it is expected that a higher spectral resolution can be obtained if low frequency cochlear implant stimulation occurs at peripheral neurons at the level of the BM instead of the SG. One idea may be to extend insertion of CI more apically, even though this may interfere with soft tissue preservation. Nonetheless, to receive accurate frequency-place stimulation with CI, variances in BM band length should be taken into account. Furthermore, angle-to-frequency correlations can enhance place pitch assessments of cochlear implant contacts according to the neural frequency bands^34,35^. Previous relationships derived between angular depth and characteristic BM frequencies have relied upon assumptions of the total angular length of cochleae^8^, and in future work the results presented herein may be used to derive angle-to-frequency relationships that are individualized based on specific BM angular length. As CI electrode positions are clearly visible in clinical CT, angle measuring would allow clinicians to obtain individualized cochlear implant programming plans using clinically obtainable values.

This is the first study to determine 3D SG tonotopy through direct dendrite tracing in intact cochleae using SR-PCI data. Tonotopic distributions of the BM and SG were assessed with respect to linear and angular length, and a Greenwood-like function was fit to relate proportional length along the SG to the associated tonotopic frequency.

## Supplementary Information

Supplementary Information.
