# Supplementary material for: Three-dimensional tonotopic mapping of the human cochlea based on synchrotron radiation phase-contrast imaging
Source: Sci Rep. 2021 Feb 24;11:4437. doi: 10.1038/s41598-021-83225-w (PMC7904830; doi:10.1038/s41598-021-83225-w)

**Supplementary material (Title page)**

**Three-dimensional tonotopic mapping of the human cochlea based on synchrotron radiation phase-contrast imaging**

Hao Li^1^, Luke Helpard ^2^, Jonas Ekeroot^1^, Seyed Alireza Rohani^3^, Ning Zhu^4^, Helge Rask-Andersen^1*^, Hanif M. Ladak^2, 3, 5*^ and Sumit Agrawal^2, 3, 5*^

*Hao Li, Ph.D., Department of Surgical Sciences, Section of Otolaryngology, Uppsala University Hospital, SE-751 85, Uppsala, Sweden. E-mail:* [*hao.li@surgsci.uu.se*](mailto:hao.li@surgsci.uu.se)

*Luke Helpard, BESc, School of Biomedical Engineering, Western University, 1152 Richmond St, London, ON, N6A 3K7, Canada. E-mail:* [*lhelpard@uwo.ca*](mailto:lhelpard@uwo.ca)

*Jonas Ekeroot, Ph.D., Department of Otolaryngology, Uppsala University Hospital, SE-751 85, Uppsala, Sweden. E-mail:* [*jonas.ekeroot@akademiska.se*](mailto:jonas.ekeroot@akademiska.se)

*Seyed Alireza Rohani, Ph.D., Department of Otolaryngology—Head and Neck Surgery, Western University, London, Ontario, Canada. E-mail:* [*srohani4@uwo.ca*](mailto:srohani4@uwo.ca)

*Ning Zhu, Ph.D., Bio‐Medical Imaging and Therapy Facility, Canadian Light Source Inc., Saskatoon, Saskatchewan, Canada. E-mail: ning.zhu@lightsource.ca*

*Helge Rask-Andersen, MD, Ph.D. Department of Surgical Sciences, Section of Otolaryngology, Uppsala University Hospital, SE-751 85, Uppsala, Sweden. E-mail:* [*helge.rask-andersen@surgsci.uu.se*](mailto:helge.rask-andersen@surgsci.uu.se)

*Hanif M. Ladak, Ph.D., Department of Otolaryngology-Head and Neck Surgery, Department of Medical Biophysics and Department of Electrical and Computer Engineering, Western University, London, ON, Canada. E-mail:* [*hladak@uwo.ca*](mailto:hladak@uwo.ca)

*Sumit Agrawal, M.D., Department of Otolaryngology-Head and Neck Surgery, Western University, London, ON, Canada. E-mail: Sumit.Agrawal@lhsc.on.ca*

**Co-senior authors.*

^1^Department of Surgical Sciences, Section of Otolaryngology, Department of Otolaryngology, Uppsala University Hospital, Uppsala, Sweden

^2^School of Biomedical Engineering, Western University, London, Ontario, Canada

^3^Department of Otolaryngology—Head and Neck Surgery, Western University, London, Ontario, Canada

^4^Bio-Medical Imaging and Therapy Facility, Canadian Light Source Inc., University of Saskatchewan, Saskatoon, Saskatchewan, Canada

^5^Department of Medical Biophysics and Department of Electrical and Computer Engineering, Western University, London, Ontario, Canada

***Corresponding authors****:*

*^1^Helge Rask-Andersen, MD, Ph.D. Department of Surgical Sciences, Section of Otolaryngology, Uppsala University Hospital, SE-751 85, Uppsala, Sweden. E-mail helge.rask-andersen@surgsci.uu.se*

*^1^Hao Li, Ph.D., Department of Surgical Sciences, Section of Otolaryngology, Uppsala University Hospital, SE-751 85, Uppsala, Sweden. E-mail:* [*hao.li@surgsci.uu.se*](mailto:hao.li@surgsci.uu.se)


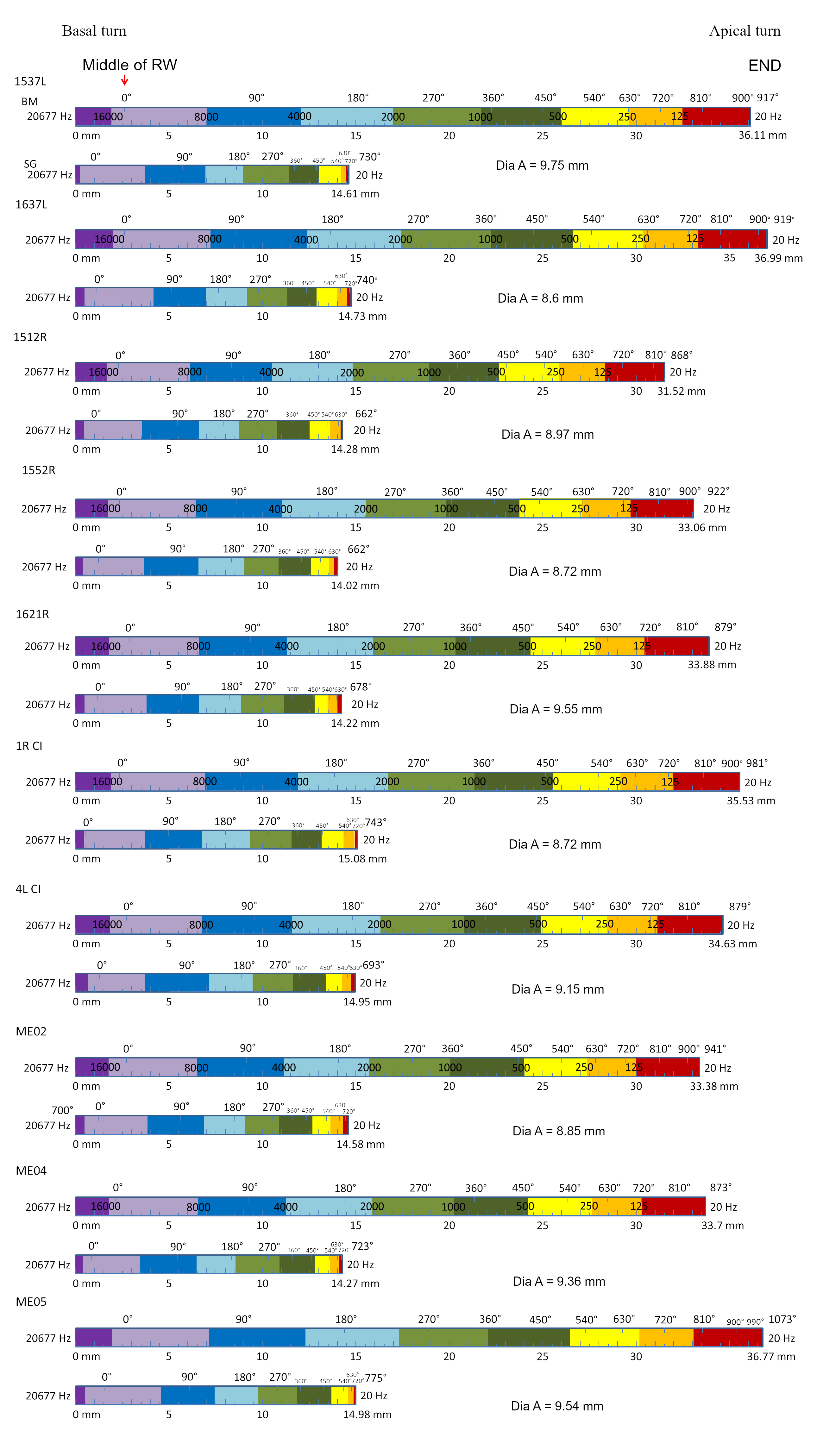

Supplement: Supplementary file 1 — Supplementary Information. [file 41598_2021_83225_MOESM1_ESM.docx]
